# Intraocular complement activation is independent of systemic complement activation and is related to macular vascular remodelling in retinal vein occlusion

**DOI:** 10.1186/s12886-024-03781-3

**Published:** 2024-11-25

**Authors:** Hengwei Liu, Yufan Zhou, Jinyan Qi, Shengnan Liang, Tingting Guo, Juan Chen, Huanhuan Tan, Jie Wang, Heping Xu, Zhongping Chen

**Affiliations:** 1 Aier Academy of Ophthalmology, Central South University, Changsha, Hunan Province 410083 China; 2 Changsha Aier Eye Hospital, Changsha, Hunan Province 410015 China; 3 Aier Eye Hospital, Jinan University, Guangzhou, Guangdong Province 510632 China; 4 School of Stomatology and Ophthalmology, Xianning Medical College, Hubei University of Science and Technology, Xianning, Hubei Province 437100 China; 5 Aier Eye Institute, Changsha, Hunan Province 410015 China; 6 Wellcome-Wolfson Institute for Experimental Medicine, School of Medicine, Dentistry and Biomedical Sciences, Queen’s University Belfast, Belfast, BT9 7BL UK

**Keywords:** Aqueous humour, Retinal vein occlusion, Complement proteins, Classical pathway, Optical coherence tomographic angiography

## Abstract

**Background:**

Retinal vein occlusion (RVO) is a major cause of vision loss. The pathogenesis remains poorly defined although inflammation is known to play a critical role. In this study, we investigated the levels of complement proteins in the aqueous humour and plasma of RVO participants and the relationship between complement levels and retinal pathologies.

**Methods:**

The plasma and aqueous humour were collected from 20 treatment-naive RVO and 20 cataract patients. Retinal lesions were examined by fundus stereoscopy and optical coherence tomography angiography. The levels of C1q, C2, C4, C4b, C3, C3b/iC3b, C5, C5a, CFB, CFD, CFI, CFH, and MBL in the plasma and aqueous humour were measured using the Luminex^®^ x MAP^®^ multiplex assay.

**Results:**

RVO patients had significantly higher levels of C4, C4b, C3b/iC3b, CFB, and CFH in the plasma and aqueous humour compared to controls. The aqueous levels of C1q, C2, C5, C5a, and MBL were also significantly higher in RVO patients than in controls. CRVO patients had higher intraocular levels of C1q, C4, C5, CFI, CFH, and MBL than BRVO patients. C5a was below the detectable limit in the plasma in 18 cataracts and 16 RVO participants. The intraocular levels of C5a positively correlated with C1q, C2, C4, C3, C5, CFB, CFH, and MBL. The intraocular levels of CFD, CFI and MBL positively correlated with CRT, and the levels of C2, C3, C5, CFB, and MBL negatively correlated with the size of the foveal avascular zone. The plasma levels of C4b, C3b/iC3b, and CFD positively correlated with their counterparts in the aqueous humour in cataracts but not in RVO patients. The ratios of aqueous humour/plasma of C1q, C4, C4b, C3b/iC3b, C5, CFB, CFD, CFI, and CFH in the RVO patients were significantly higher than those in the cataract patients.

**Discussion and conclusions:**

The intraocular complement activation in RVO is mediated by the classical and the alternative pathways and is largely independent of systemic complement activation. Intraocular complement activation may be related to retinal oedema and vascular remodeling in RVO patients.

## Background

Retinal vein occlusion (RVO), a relatively common vascular disorder of the retina, represents one of the leading causes of vision loss globally. There are two types-branch retinal vein occlusion (BRVO) and central retinal vein occlusion (CRVO), affecting primarily individuals with advancing age, diabetes, arteriosclerosis, hypertension, hyperlipidemia, and blood hyperviscosity [1]. Vision loss in RVO is chiefly attributed to retinal ischemia, subsequent microvascular degeneration, and macular oedema. Contemporary treatment approaches include the intravitreal injection of vascular endothelial cell growth factor (VEGF) inhibitors and laser photocoagulation [2]. Despite the reported success of these treatments, 46–72% of RVO patients continue to experience refractory or recurring macular oedema, highlighting the multifaceted nature of RVO pathogenesis [3].

Inflammation is known to play an important role in vascular dysfunction-mediated macular oedema including diabetic retinopathy, age-related macular degeneration, and RVO [4]. Higher intraocular levels of inflammatory mediators including cytokines (e.g., IL-8, IL-6, and CCL2) [5], metalloproteinase [6] and neutrophil elastase (e.g., lipocalin-2) [7] have been observed in RVO patients. Some inflammatory mediators are related to RVO-mediated retinal pathologies and the response to anti-VEGF therapy [6, 7]. The complement system constitutes an important part of innate immunity. Apart from the cell-killing roles of the membrane attack complex (C5b-9), complement fragments are critically involved in phagocytosis (e.g., C3b-mediated opsonizing antigens) and inflammation (e.g., C3a- and C5a- mediated immune cell recruitment and activation). Dysregulated complement activation is known to be involved in a variety of retinal diseases including uveitis, diabetic retinopathy, and age-related macular degeneration [8–10]. Higher aqueous levels of C3, C5 and complement factor H (CFH) have been reported in RVO patients [11], although the link between the intraocular complement system and RVO-related retinal pathologies remains elusive. The complement system is actively involved in the thrombosis process. It is unclear whether uncontrolled intraocular complement activation is a localized tissue-level event or a part of systemic/circulatory abnormal complement activation in RVO.

The complement system can be activated through the classical pathway (CP), the mannose-binding lectin (MBL) pathway, and the alternative pathway (AP). The AP is also critically involved in amplifying the complement activation cascade by forming the C3-convertase (C3bBb) and C5-convertase (C3bBbC3b).

In this study, we aimed to understand (1) whether the development of RVO is related to abnormal complement activation either systemically or locally inside the eye, and (2) the link between intraocular complement activation and RVO-related retinal pathologies. To achieve the goals, we measured the levels of thirteen complement proteins/fragments, including 4 proteins in the CP (C1q, C2, C4, and C4b), 4 factors in the AP (CFB, CFD, CFI, and CFH), 1 protein in the MBL pathway (MBL), and 4 proteins involved in the common pathway (C3, C3b/iC3b, C5, and C5a) in the plasma and the aqueous humour of 20 RVO and 20 cataract patients. We further investigated the relationship between the complement levels and clinical presentations of RVO.

## Methods

### Participants and sample collection

The study was conducted under the Declaration of Helsinki and the study protocol was approved by the Institutional Review Board (IRB) of Changsha Aier Eye Hospital (Ethical approval number: (2020) KYPJ005). Informed consent was obtained from all participants. The sample size was estimated by using an online clinical sample size calculator (https://clincalc.com/stats/samplesize.aspx) based on the levels of C3 fragments (C3a, C3b/iC3b) in the plasma [12] and aqueous humour [13] in neovascular age-related macular degeneration (AMD), myopic retinopathy, and healthy controls reported in our previous studies. To detect similar levels of change in the plasma and aqueous humor complement levels in RVO patients with 5% probability and 80% power, 30 plasma samples and 21 aqueous humour samples in each group will be needed. Due to various restrictions including the COVID-19 pandemic, we were only able to recruit 20 treatment-naive RVO patients (6 with CRVO and 14 with BRVO) and 20 senile cataract patients were recruited for this study.

The inclusion and exclusion criteria have been described in our previous study [14]. In brief, patients who were confirmed to have RVO and did not receive any treatment, but require medical attention were recruited to the study. Senile cataract patients who underwent phacoemulsification and lens implantation were enrolled as controls. The exclusion criteria were patients with (1) other ocular diseases; (2) a history of intraocular or systemic inflammation or autoimmune diseases or the use of immune suppressive medications such as steroids; (3) cancer; (4) a history of ocular surgery (including laser treatment) within 6 months. For participants with cataract surgery, if fundus examination was not possible, the patients were excluded from the study.

Fasting blood samples (5 mL) were collected into a purple sterile vacuum tube with EDTAK2 anti-coagulant (Huabo Medical Care, Heze, Shandong, China) and were rotated several times immediately before centrifugation for plasma collection. 60 µL of aqueous humour were collected from RVO patients immediately before their first intraocular injection of VEGF inhibitors or steroids. The same amount of aqueous humour was collected from cataract patients during surgery. All samples were stored at -80 °C until laboratory analysis. The time between sample collection and sample storage was less than 30 min.

### Clinical data collection

All participants received thorough clinical evaluations (including optical coherence tomography angiography, OCTA), which were detailed in our previous publications [14]. Below parameters were collected for further analysis in this study: best corrected visual acuity (BCVA, logMAR); the overall size of foveal avascular zone (FAZ); perimeter of foveal avascular zone (PERIM); central retinal thickness (CRT); inner limiting membrane-inner plexiform layer thickness (ILM-IPL); superficial capillary plexus vessel density (SVD); and deep capillary plexus vessel density (DVD). RVO patients with CRT greater than 300 μm and the logMAR best corrected visual acuity greater than 0.3 were considered to have the indications for intravitreal injection of the vascular endothelial cell growth factor (VEGF) inhibitor (Ranibizumab). Dexamethasone (Ozurdex) was used for patients with intraocular lens implanted previously or whose medical insurance does not cover VEGF inhibitors (to reduce financial burden).

### Measurement of complement proteins/factors

The levels of C1q, C2, C4, C4b, C3, C3b/iC3b, C5, C5a, CFB, CFD, CFI, CFH and MBL in aqueous humour and plasma samples were measured using the Luminex^®^ x MAP^®^ technology following manufacturer’s instructions (Luminex xMap Technology, Bio-Rad, Shanghai, China). The aqueous humour and plasma were diluted 20- and 200-times respectively and a total of 25 µL of diluted sample was used from each participant in the study. The fluorescent intensity in each well in the plates was measured using a plate reader with the Luminex^®^ x PONENT^®^ acquisition software (MAGPIX^®^). The concentrations of complement proteins were calculated using the MILLIPLEX^®^ Analyst 5.1 (MAGPIX^®^).

### Statistical analysis

All data were analyzed using the Statistical Package for Social Sciences (SPSS, V.24.0) software. The chi-square test was used to compare categorical variables. The Mann–Whitney U test was used to compare values between two groups. The difference between three groups was analyzed using the Kruskal-Wallis test followed by Bonferroni correction. The differences between groups were calculated using covariance analysis adjusted by age. The correlation between complement proteins and clinical parameters was assessed using the Spearman correlation test. *P* < 0.05 was considered statistically significant.

## Results

### Clinical characteristics

There was no significant difference in gender distribution, history of hypertension or diabetes, history of smoking, BMI, or BCVA between RVO patients and controls (Table 1). The average age of RVO patients was significantly younger than controls (59.00 years vs. 66.50 years) (Table 1). The central retinal thickness (CRT) of RVO (both CRVO and BRVO) patients was significantly higher than controls (550.00 μm, 628.00 μm, 516.00 μm vs. 220.00 μm) (Table 1). Age, CRT, foveal avascular zone (FAZ), perimeter (PERIM), inner limiting membrane-inner plexiform layer (ILM-IPL) thickness, superficial vessel density (SVD) and deep vessel density (DVD) did not differ between BRVO and CRVO.

**Table 1:** Demographic and clinical characteristics of study patients

Patients characteristics	Control *N* = 20	RVO *N* = 20	Subgroups of RVO		
			CRVO *N* = 6	BRVO *N* = 14	*P*-values BRVO vs. CRVO
Age, median (Q1;Q3)	66.50 (63.50; 70.00)	59.00 (54.50; 67.00)*^a^	58.50 (57.00; 63.00)	59.00 (52.00; 69.00)	0.968
Female (%)^b^	50	60	83.33	50	0.163
Hypertension (%)^b^	40	55	16.66	71.43	0.111
Diabetes (%)^b^	10	10	16.66	7.14	0.515
Smoking (%)^b^	25	15	16.66	14.29	0.891
BMI, median (Q1;Q3), kg/m^2^	22.97 (19.57; 25.46)	24.34 (22.18; 27.51)	23.89 (21.30; 24.34)	25.51 (23.05; 28.13)	0.274
BCVA, median (Q1;Q3)	0.52 (0.35; 1.35)	0.92 (0.65; 1.05)	1.00 (1.00; 1.10)	0.92 (0.60; 1.00)	0.312
CRT, median (Q1;Q3)	220.00(198.50;237.50)	550.00 (454.50;660.00)**^a^	628.00 (573.00;778.00)**^c^	516.00 (425.00;595.00)**^c^	0.179
FAZ, median (Q1;Q3)	\	0.22 (0.12; 0.32)	0.21 (0.12; 0.33)	0.22 (0.11; 0.30)	0.841
PERIM, median (Q1;Q3)	\	2.07 (1.43; 2.24)	2.17 (1.38; 2.29)	1.93 (1.46; 2.17)	0.659
ILM-IPL, median (Q1;Q3)	\	89.00 (68.00; 149.81)	118.31 (83.00; 265.05)	89.00 (59.14; 131.00)	0.353
SVD, median (Q1;Q3)	\	45.00 (43.75; 47.55)	44.70 (41.00; 46.90)	45.05 (44.40; 47.60)	0.602
DVD, median (Q1;Q3)	\	44.45 (43.30; 47.23)	44.88 (44.00; 48.00)	44.10 (40.70; 46.46)	0.353

* *p* < 0.05; ** *p* < 0.01

^a^, Mann–Whitney U test analyzed controls and RVO patients; ^b^, Chi-square test; ^c^, Kruskal-Wallis test analyzed controls, CRVO, and BRVO patients followed by Bonferroni correction

BMI: body mass index; BCVA: best corrected visual acuity, logMAR; CRT: central retinal thickness; FAZ: foveal avascular zone; PERIM: perimeter of foveal avascular zone; ILM-IPL: inner limiting membrane-inner plexiform layer; SVD: superficial capillary plexus vessel density; DVD: deep capillary plexus vessel density; BRVO: branch retinal vein occlusion; CRVO: central retinal vein occlusion; RVO: retinal vein occlusion

### Levels of complement proteins in the aqueous humour

Out of the 13 complement proteins, C3b/iC3b was below the detectable level (< 8.02 ng/ml) in 3 cataracts and 2 RVO patients, C5a was detected in 4 cataract patients (from 1.65 ~ 18.91 pg/ml) but in all RVO patients. C5 was below the detectable level (< 0.52 ng/ml) in 2 cataract patients. Other complement proteins were detected in the aqueous humour in all participants. After adjusting for age, the levels of C1q, C2, C4, C4b, C3b/iC3b, C5, C5a, CFB, CFH, and MBL in the RVO group were significantly higher than those in the control group (Table 2). The level of C3 did not differ between the two groups (Table 2). Further subgroup analyses of RVO patients showed that the levels of C1q, C2, C4, C4b, C3b/iC3b, C5, C5a, CFB, CFD, CFI, CFH, and MBL in the CRVO patients were significantly higher than those in controls (Table 2). The levels of C1q, C2, C4, C4b, C3b/iC3b, C5a, CFB, CFH, and MBL in the BRVO patients were also significantly higher than those in the controls after adjusting for age (Table 2). Furthermore, patients with CRVO had significantly higher levels of C1q, C4, C5, CFI, CFH, and MBL than patients with BRVO (Table 2). Our results may suggest that the intraocular levels of complement proteins are related to the severity of RVO.

**Table 2:** Aqueous humour levels of complement proteins/factors in RVO patients and controls

			Subgroups of RVO		
Variables median (Q1;Q3)	Control	RVO^a^	CRVO^c^	BRVO^c^	*P*-values
	(*n* = 13)	(*n* = 19)	(*n* = 5)	(*n* = 14)	BRVO vs. CRVO^b^
C1q(ng/mL)	2.04 (1.34;4.34)	12.34 (5.62;19.70)**	16.47 (16.26;20.75)**	8.59 (4.77;14.61)**	**0.034**
C2(ng/mL)	3.68 (3.02;5.28)	34.75 (23.46;77.60)**	61.01 (34.75;77.60)**	33.64 (22.36;73.73)**	0.343
C4(ng/mL)	458.48 (280.26;589.80)	980.32 (788.49;1131.00)**	1128.00 (1060.58;1150.00)**	868.24 (730.76;1013.00)**	**0.026**
C4b(ng/mL)	70.27 (50.92;93.11)	124.68 (103.84;131.25)**	124.68 (119.05;131.25)**	124.35 (73.27;101.16)*	0.500
C3(ng/mL)	154.76 (116.00;173.74)	180.92 (138.76;179.94)	138.76 (104.92;185.34)	185.81 (99.30;131.94)	0.298
C3b/iC3b(ng/mL)	15.83 (10.25;20.28)	187.54 (122.34;410.28)**	472.58 (135.62;508.07)**	175.74 (152.34;219.15)**	0.257
C5(ng/mL)	2.25 (0.68;2.48)	11.46 (5.14;29.59)*	32.54 (29.59;41.40)**	7.48 (1.82;13.06)	**0.005**
C5a(pg/mL)	1.14 (1.14;2.56)	11.46 (5.14;29.59)**	60.90 (50.84;107.78)**	7.48 (1.82;13.06)*	0.156
CFB(ng/mL)	186.34 (110.55;276.14)	380.21 (257.06;531.40)**	380.21 (364.31;603.14)**	352.79 (207.64;494.82)*	0.257
CFD(ng/mL)	48.47 (42.90;55.59)	64.20 (52.67;71.44)	71.44 (65.68;88.50)*	58.04 (51.71;67.38)	0.070
CFI(ng/mL)	34.77 (27.35;88.94)	84.12 (59.69;185.17)	229.89 (165.75;259.30)**	66.47 (44.75;91.31)	**0.005**
CFH(ng/mL)	71.26 (51.77;76.57)	320.76 (218.34;572.41)**	665.26 (325.59;674.02)**	267.73 (216.35;352.33)**	**0.044**
MBL(ng/mL)	0.16(0.11;0.21)	0.68(0.28;1.00)**	1.09(1.00;1.02)**	0.45(0.28;0.71)**	**0.005**

* *p* < 0.05; ** *p* < 0.01. Bold indicating p value was statistically significant

^a^, Covariance analysis of complement proteins/factors between controls and RVO patients after adjusting for age; ^b^, Mann–Whitney U test analysis between BRVO and CRVO patients; ^c^, Covariance analysis of complement proteins/factors between controls and CRVO, BRVO patients after adjusting for age

RVO: retinal vein occlusion; BRVO: branch retinal vein occlusion; CRVO: central retinal vein occlusion; CFB: complement factor B; CFD: complement factor D; CFI: complement factor I; CFH: complement factor H; MBL: mannose-binding lectin

### Levels of complement proteins in the plasma

In the 40 plasma samples, C5a was below the detectable limit in 18 cataract and 16 RVO patients. Therefore, C5a was not included in further analysis. Other complement proteins were detected in all samples. After adjusting for age, the levels of C4, C4b, C3b/iC3b, CFB, and CFH in the RVO group were significantly higher than those in the control group (Table 3). The levels of C1q, C2, C3, C5, CFD, CFI, and MBL did not differ between the two groups (Table 3). Further subgroup analyses of RVO patients showed that the levels of C3b/iC3b and CFI in the CRVO patients were significantly higher than those in the controls (Table 3). In addition, the levels of C2, C4, C4b, and C3b/iC3b in the BRVO patients were also significantly higher than those in the controls (Table 3). Moreover, patients with CRVO had significantly higher levels of CFI than patients with BRVO (Table 3). Our results suggest that RVO patients may have higher levels of systemic complement activation.

**Table 3:** Plasma levels of complement proteins/factors in RVO patients and controls

			Subgroups of RVO		
Variables median (Q1;Q3)	Control	RVO^a^	CRVO^c^	BRVO^c^	*P*-values
	(*n* = 20, ×10^3^ng/mL)	(*n* = 20, ×10^3^ng/mL)	(*n* = 6, ×10^3^ng/mL)	(*n* = 14, ×10^3^ng/mL)	BRVO vs. CRVO^b^
C1q	29.55 (28.03;36.45)	34.41 (28.39;40.75)	35.32 (28.44;40.31)	34.41 (28.34;42.28)	0.779
C2	0.98 (0.85;2.23)	7.48 (1.75;40.13)	3.27 (1.55;10.28)	8.95 (1.95;15.25)*	0.547
C4	507.73 (430.96;576.00)	654.14 (592.35;1459.17)**	622.29 (563.67;729.56)	681.05 (592.65;788.78)**	0.494
C4b	20.91 (17.38;25.60)	27.78 (59.24;75.09)*	25.90 (20.27;28.34)	28.20 (25.04;33.35)*	0.239
C3	46.32 (34.73;52.77)	28.26 (21.83;30.36)	42.57 (21.70;63.54)	28.26 (21.97;68.52)	0.904
C3b/iC3b	40.75 (31.11;50.24)	106.66 (72.86;161.01)**	109.93 (59.10;184.98)**	106.66 (81.80;127.99)**	0.904
C5	10.81 (0.92;12.40)	11.53 (10.16;13.77)	13.25 (10.25;13.84)	10.83 (10.07;13.71)	0.547
CFB	15.81 (14.18;17.98)	182.12 (161.52;220.99)*	196.29 (164.12;226.83)	175.81 (158.92;215.16)	0.718
CFD	6.23 (5.41;7.07)	5.84 (4.85;7.11)	5.64 (5.04;5.92)	6.29 (4.81;7.58)	0.494
CFI	22.89 (9.87;26.80)	26.19 (22.45;33.04)	34.31 (26.98;37.38)*	23.12 (10.70;28.36)	**0.015**
CFH	204.98 (191.73;232.19)	226.95 (206.79;247.66)*	231.90 (223.37;253.39)	215.19 (199.54;241.93)	0.444
MBL	2.05 (1.04;4.77)	3.45 (1.20;6.09)	4.10 (1.53;5.39)	3.45 (1.18;6.78)	0.968

* *p* < 0.05; ** *p* < 0.01. Bold indicating p value was statistically significant

^a^, Covariance analysis of complement proteins/factors between controls and RVO patients after adjusting for age; ^b^, Mann–Whitney U test analysis between BRVO and CRVO patients; ^c^, Covariance analysis of complement proteins/factors between controls and CRVO, BRVO patients after adjusting for age

RVO: retinal vein occlusion; BRVO: branch retinal vein occlusion; CRVO: central retinal vein occlusion; CFB: complement factor B; CFD: complement factor D; CFI: complement factor I; CFH: complement factor H; MBL: mannose-binding lectin

### The relationship between aqueous humour C3b/iC3b, C5a and other complement proteins in RVO patients

Complement fragments C3b/iC3a and C5a are generated during complement activation. To understand which of the complement pathways are involved in the cleavage of C3 and C5 in the RVO retina, we examined the relationship of these C3b/iC3b, C5a and other complement proteins. Spearman correlation analysis showed that C3b/iC3b had intermediate correlations with C1q, C2, C4, C4b, C5, and CFH (*r* = 0.456 ~ 0.656), indicating that the CP and AP complement system may be involved in intraocular C3 cleavage in RVO. C5a positively correlated with C1q (*r* = 0.533), C2 (*r* = 0.789), C4(*r* = 0.644), C3 (*r* = 0.502), C5 (*r* = 0.877), CFB (*r* = 0.632), CFH (*r* = 0.795), and MBL (*r* = 0.611) (Table 4). Interestingly, the AP-related complement regulators, CFB and CFH, positively correlated with C1q, C2, and C4. Our results suggest that the final step of intraocular complement activation (i.e., breakdown of C5) may be initiated through the CP in RVO, but amplified by the AP.

**Table 4:** Correlation between aqueous humour complement proteins/factors and visual acuity and OCTA parameters in RVO patients

	C1q	C2	C4	C4b	C3	C3b/iC3b	C5	C5a	CFB	CFD	CFI	CFH	MBL	VA	FAZ	PERIM	CRT	ILM-IPL	SVD	DVD
C1q	/	0.656**	0.805**	0.542*	0.137	0.656**	0.731**	0.533*	0.570*	0.407	0.626**	0.782**	0.638**	0.282	-0.251	-0.067	0.135	0.188	0.088	-0.218
C2	0.002	/	0.672**	0.328	0.554*	0.489*	0.824**	0.789**	0.825**	0.453	0.333	0.853**	0.637**	0.004	-0.547*	-0.349	0.042	0.079	0.278	-0.254
C4	0.000	0.002	/	0.665**	0.181	0.474*	0.801**	0.644**	0.568*	0.447	0.540*	0.851**	0.496*	0.240	-0.063	0.212	0.127	0.276	0.040	-0.181
C4b	0.016	0.170	0.002	/	-0.096	0.456*	0.319	0.179	0.056	0.505*	0.507*	0.484*	0.263	0.160	0.121	0.486*	0.187	0.393	0.152	-0.032
C3	0.576	0.014	0.459	0.694	/	-0.091	0.318	0.502*	0.556*	0.146	-0.098	0.346	0.201	-0.347	-0.528*	-0.404	-0.126	-0.313	0.143	-0.439
C3b/iC3b	0.002	0.033	0.040	0.050	0.710	/	0.482*	0.340	0.235	0.116	0.279	0.563*	0.428	0.401	-0.198	-0.168	-0.180	0.100	0.124	0.121
C5	0.000	0.000	0.000	0.183	0.184	0.037	/	0.877**	0.769**	0.499*	0.426	0.919**	0.722**	0.209	-0.472*	-0.167	0.053	0.195	-0.015	-0.120
C5a	0.019	0.000	0.003	0.464	0.029	0.154	0.000	/	0.632**	0.407	0.144	0.795**	0.611**	0.133	-0.442	-0.146	-0.008	0.257	0.126	-0.098
CFB	0.011	0.000	0.011	0.819	0.013	0.333	0.000	0.004	/	0.275	0.251	0.709**	0.493*	0.068	-0.561*	-0.389	-0.086	-0.24	0.069	-0.377
CFD	0.084	0.052	0.055	0.027	0.552	0.637	0.030	0.084	0.254	/	0.477*	0.535*	0.673**	0.059	-0.242	0.137	0.484*	0.468*	0.350	0.041
CFI	0.004	0.163	0.017	0.027	0.689	0.247	0.069	0.557	0.300	0.039	/	0.461*	0.706**	0.000	-0.202	-0.053	0.617**	0.355	-0.241	-0.230
CFH	0.000	0.000	0.000	0.036	0.147	0.012	0.000	0.000	0.001	0.018	0.047	/	0.699**	0.204	-0.430	-0.181	0.154	0.158	0.121	-0.190
MBL	0.003	0.003	0.031	0.276	0.409	0.067	0.000	0.005	0.032	0.002	0.001	0.001	/	0.107	− 0.571*	-0.327	0.471*	0.386	0.073	-0.056
VA	0.242	0.989	0.323	0.512	0.146	0.089	0.391	0.588	0.782	0.809	1.000	0.401	0.664	/	0.030	0.121	-0.006	-0.021	-0.003	0.108
FAZ	0.300	0.015	0.797	0.622	0.020	0.416	0.041	0.058	0.012	0.318	0.408	0.066	0.011	0.899	/	0.728**	0.019	0.084	0.133	0.286
PERIM	0.786	0.143	0.383	0.035	0.087	0.491	0.494	0.552	0.099	0.576	0.831	0.459	0.171	0.612	0.000	/	0.029	0.326	0.204	0.170
CRT	0.581	0.864	0.604	0.444	0.606	0.461	0.830	0.974	0.726	0.036	0.005	0.528	0.042	0.978	0.937	0.902	/	0.462*	0.134	-0.167
ILM-IPL	0.441	0.748	0.252	0.096	0.192	0.684	0.423	0.288	0.321	0.043	0.136	0.518	0.102	0.932	0.726	0.161	0.040	/	0.235	0.287
SVD	0.720	0.249	0.869	0.534	0.560	0.613	0.951	0.608	0.780	0.141	0.320	0.623	0.766	0.991	0.577	0.389	0.574	0.318	/	0.195
DVD	0.371	0.295	0.459	0.898	0.060	0.621	0.626	0.689	0.111	0.867	0.344	0.437	0.821	0.651	0.222	0.474	0.481	0.221	0.409	/

*P ≤ 0.05, **p ≤ 0.01; The value above the diagonal empty cell (/) represents the Spearman correlation coefficient. The value below the diagonal empty cell (/) indicates the level of statistical significance of the correlation. CFB: complement factor B; CFD: complement factor D; CFI: complement factor I; CFH: complement factor H; MBL: mannose-binding lectin; VA: visual acuity; FAZ: foveal avascular zone; PERIM: perimeter of foveal avascular zone; CRT: central retinal thickness; ILM-IPL: inner limiting membrane-inner plexiform layer; SVD: superficial capillary plexus vessel density; DVD: deep capillary plexus vessel density; RVO: retinal vein occlusion

### The relationship between aqueous humour complement proteins and OCTA parameters in RVO patients

To understand the link between intraocular complement activation and retinal pathology in RVO, we further investigated the relationship between intraocular complement proteins and retinal OCTA parameters. The aqueous levels of CFD, CFI and MBL positively correlated with the central retinal thickness (CRT). CFD also positively correlated with the thickness of the inner limiting membrane-inner plexiform layer (ILM-IPL). The levels of C2, C3, C5, CFB, and MBL negatively correlated with the size of the foveal avascular zone (FAZ). There is a moderate correlation between C4b levels and the perimeter of the foveal avascular zone (PERIM) (Table 4). Surprisingly, we did not find any correlation between complement fragments (C3b/iC3b, C4b, and C5a) and OCTA parameters. No correlation was observed between the aqueous humour levels of complement proteins and visual acuity, the vascular density of the superficial, and deep capillary plexus (Table 4).

### Correlation between aqueous humour and plasma complement proteins

To understand if intraocular complement proteins are affected by the circulating complement system, we investigated the relationship between plasma and aqueous humour complement proteins. In cataract patients, moderate to strong positive correlations (*r* = 0.57 ~ 0.74) were observed in C4b, C3b/iC3b, CFD, and CFI (Table 5). Surprisingly, in RVO patients, only CFI in the aqueous humour positively correlated with that in the plasma (Table 5). We further investigated the ratios of aqueous humour/plasma of complement proteins in RVO and cataract controls (Table 6). After adjusting for age, the ratios of C1q, C4, C4b, C3b/iC3b, C5, CFB, CFD, CFI, and CFH in RVO patients were markedly higher than in cataract controls (Table 6). Subgroup analysis showed that the ratios of C1q, C4, C5, CFD, and CFH in CRVO were significantly higher than those in BRVO (Table 6). Our results suggest that the intraocular complement activation in RVO patients is largely independent of the circulating complement system.

**Table 5:** Correlation analysis of complement proteins/factors in the plasma and the aqueous humour

Variables	Control		RVO	
	*r*	*p*	*r*	*p*
C1q	-0.172	0.575	0.061	0.803
C2	-0.006	0.986	-0.126	0.606
C4	0.544	0.055	0.047	0.847
C4b	0.725**	0.005	0.293	0.223
C3	-0.172	0.575	-0.039	0.875
C3b/iC3b	0.566*	0.044	0.105	0.668
C5	-0.324	0.280	0.397	0.092
CFB	-0.209	0.494	0.170	0.486
CFD	0.736**	0.004	0.444	0.057
CFI	0.670*	0.012	0.609**	0.006
CFH	0.280	0.354	0.251	0.300
MBL	-0.590	0.034	0.198	0.415

Correlations were calculated as Spearman correlation coefficient (r). *P ≤ 0.05, **p ≤ 0.01. CFB: complement factor B; CFD: complement factor D; CFI: complement factor I; CFH: complement factor H; MBL: mannose-binding lectin; RVO: retinal vein occlusion

**Table 6:** The ratio of complement proteins/factors in the aqueous humour to those in the plasma in RVO patients and controls

			Subgroups of RVO		
Variables median (Q1;Q3)	Control	RVO^a^	CRVO^c^	BRVO^c^	*P*-values
	(*n* = 13, ×10^− 3^)	(*n* = 19, ×10^− 3^)	(*n* = 5, ×10^− 3^)	(*n* = 14, ×10^− 3^)	BRVO vs. CRVO^b^
C1q	0.077(0.04;0.11)	0.336(0.172;0.5)**	0.500(0.42;0.767)**	0.244(0.11;0.4)*	**0.014**
C2	2.995(1.569;4.739)	4.493(1.945;20.509)	5.142(3.897;47.316)	4.213(1.6;18.037)	0.391
C4	0.959(0.5;1.168)	1.351(1.046;1.776)**	2.040(1.335;2.242)**	1.259(0.98;1.54)	**0.044**
C4b	3.324(2.454;3.829)	4.262(3.538;5.313)**	5.576(4.047;6.573)*	4.116(3.372;5.17)	0.087
C3	3.399(2.064;5.942)	4.121(2.647;8.933)	2.914(2.486;75.62)	6.118(2.846;9.053)	0.559
C3b/iC3b	0.334(0.279;0.502)	1.803(1.101;2.551)**	2.523(1.094;8.913)**	1.668(1.063;2.47)**	0.343
C5	0.151(0.061;0.296)	0.944(0.471;2.887)**	3.038(1.915;5.351)**	0.606(0.195;1.262)*	**0.014**
CFB	1.386(0.675;1.849)	2.081(1.387;2.555)*	2.487(1.638;3.916)	1.928(1.258;2.457)	0.186
CFD	7.195(6.808;7.811)	10.556(8.42;12.415)**	13.027(11.549;16.301)**	10.069(8.183;10.881)	**0.007**
CFI	2.893(2.143;3.533)	4.601(2.889;7.258)*	9.068(3.784;11.062)*	3.816(2.451;5.513)	0.056
CFH	0.315(0.251;0.416)	1.237(1.029;2.601)**	2.886(1.306;3.16)**	1.175(0.994;1.633)**	**0.026**
MBL	0.172(0.031;0.471)	0.180(0.076;0.604)	0.229(0.183;0.481)	0.114(0.072;0.624)	0.298

* *p* < 0.05; ** *p* < 0.01. Bold indicating p value was statistically significant

^a^, Covariance analysis of complement proteins/factors between controls and RVO patients after adjusting for age; ^b^, Mann–Whitney U test analysis between BRVO and CRVO patients; ^c^, Covariance analysis of complement proteins/factors between controls and CRVO, BRVO patients after adjusting for age

RVO: retinal vein occlusion; BRVO: branch retinal vein occlusion; CRVO: central retinal vein occlusion; CFB: complement factor B; CFD: complement factor D; CFI: complement factor I; CFH: complement factor H; MBL: mannose-binding lectin

### Discussion and conclusions

In this study, we show that RVO patients had significantly higher plasma and aqueous levels of complement proteins compared with cataract patients. Five (C4, C4b, C3b/iC3b, CFB, and CFH) out of 13 complement proteins in the plasm were significantly higher in RVO patients compared to the controls. In aqueous humour, apart from C3, CFD, and CFI, other complement proteins were significantly higher in RVO patients. In our study, we excluded participants with inflammatory/autoimmune diseases and those who were taking immunosuppressant medications. RVO patients were younger than cataract patients, but the differences in complement proteins remained after adjusting for age. Our results suggest that complement activation, in particular intraocular complement activation, may be critically involved in RVO development and RVO-mediated retinal pathologies. Our results also suggest that CRVO patients may have higher levels of complement activation compared with BRVO. However, due to the small sample size further large cohort studies are needed to confirm the results.

The occlusion of the retinal vein/venules in RVO is due to the formation of thrombosis. Although the mechanism underlying abnormal thrombosis in RVO remains elusive, the complement and coagulation pathways are closely related. There are multiple cross-talks between the components of the two cascades. For example, plasma kallikrein can affect the generation of C3 and C5 fragments directly or indirectly [15]. C5a can be generated by thrombin independent of C3 [16]. On the other hand, complements can activate the coagulation cascade directly or indirectly. The MASP2, a component of the MBL pathway, is critically involved in the activation of thrombin and subsequent generation of the fibrin mesh [17]. Sublytic C5b-9 can cause transient membrane depolarization, granule secretion, and induction of platelet-catalyzed thrombin generation and clotting [18, 19].

Although C5a was below the detectable limit in 16 out of 20 RVO patients and this does not support systemic complement activation, the higher levels of C4 and C3b/iC3b in the plasma of RVO patients are indicatives of abnormal systemic complement activities.

Ten out of 13 complement proteins in the aqueous humour were significantly higher in RVO patients compared to controls. The complement levels in the plasma were 10-1000 times higher than those in the aqueous humour in our study. Theoretically, circulating complement proteins can leak into the retina from the diseased vessels and accumulate in the intraocular compartments. Surprisingly, we detected positive correlations between plasma and aqueous levels of four complement proteins (C4b, C3b/iC3b, CFD, and CFI, *r* = 0.57 ~ 0.74) in cataract patients, but only one (i.e., CFI, *r* = 0.609) in RVO patients. Moreover, by comparing the levels of complement proteins/factors in aqueous humour and plasma between RVO patients and the control group, after considering age factors, the concentrations of C1q, C4, C4b, C3b/iC3b, C5, CFB, CFD, CFI, and CFH in the RVO patients were significantly higher than those in the cataract patients. Our results suggest that intraocular complement proteins may be somehow, related to their counterparts in the blood circulation under normal physiological conditions, but in RVO, they are independent of their circulating counterparts and may be generated locally within the eye. In other words, intraocular complement production/activation is likely an active response of the retina to ischemic injury in RVO. The retina has a higher level of control over its immune response to injury [20]. The complement system forms an important arm of retinal innate immune protection. Retinal cells, including neurons, microglia [21], and RPE cells [22–26] express various complement genes and their expression is increased under inflammatory conditions [24]. Therefore, it is not surprising to see higher levels of complement activation in the RVO retina. In line with our study, C3, C5 and CFH were detected in the aqueous humour from RVO patients using proteomic analysis by others [11, 27].

The complement system can be activated through the CP, AP, and MBL pathways and the cleavages of C3 and C5 are two key milestone cascades. The intraocular level of C3b/iC3b positively correlated with C1q, C2, C4, C4b, C5, and CFH, indicating that all three pathways are involved in the cleavage of C3 in the RVO retina. On the other hand, the level of C5a positively correlated with C1q, C2, C4, C3, C5, CFB, CFH, and MBL in the aqueous humour of RVO patients. The levels of C2 and C4 were strongly correlated with CFB and CFD (Table 4). Our results suggest that intraocular complement activation in RVO is likely mediated through the CP and supported by the AP through amplifying the CP-mediated activation cascade. It is worth noting that C3b/iC3b but not C5a was detected in the aqueous humour of cataract patients and the intraocular level of C5a did not correlate with C3b/iC3b in RVO patients, suggesting that C3 cleavage does not necessarily lead to C5 cleavage inside the eye. Furthermore, C5a can be generated by thrombin independent of C3 [13]. Multiple pathways may be involved in the breakdown of C5 in RVO.

Dysregulated complement activation can lead to pathologies. We found that aqueous levels of CFD, CFI and MBL positively correlated with CRT. A positive correlation was also observed between the aqueous level of CFD and the ILM-IPL thickness. There is a moderate correlation between C4b levels and the perimeter of the foveal avascular zone (PERIM). Furthermore, the aqueous levels of C2, C3, C5, CFB, and MBL were negatively correlated with the size of the foveal avascular zone (FAZ). The FAZ is the most sensitive region of the retina and can indirectly reflect the alterations of macular microcirculation. In certain retinal diseases, such as diabetic retinopathy and RVO [28], the FAZ can exhibit changes in size, shape, or perfusion, which are related to macular capillary remodeling, disease progression, and the impairment of visual function [29]. Our results suggest that intraocular complement activation might be involved in retinal oedema and macular microvascular remodeling in RVO. Since we did not detect any relationship between C3b/iC3b, C5a (indicators of complement activation) and retinal OCTA parameters, intraocular complement activation per se is unlikely a contributor to macular oedema and vascular degeneration in RVO. We previously reported higher intraocular levels of inflammation cytokines and the activation of the related pathways such as PI3K-Akt, Ras, MAPK, and Jak/STAT in RVO patients [14]. Retinal thickness positively correlated with intraocular levels of Flt-3 L, IL-33, GROβ, PD-L1, G-CSF, and TGF-α [14]. The complement system constitutes an important arm of the inflammatory response and dysregulated complement activation may lead to abnormal cytokine production. Complement fragments C3a, C3b/iC3b, C5a, and the sublytic MAC are known to have immune regulatory roles. The complement system may contribute to retinal pathologies in RVO by regulating inflammation, although further studies will be needed to elucidate the mechanisms.

The strengths of the study include (1) the simultaneous measurement of complement proteins in the blood and aqueous humour; and (2) comprehensive clinical and laboratory evaluations of the same participants; (3) participants did not receive any medication (systemic or local) before the study. The study has several limitations. Firstly, the number of participants enrolled in this study was relatively small. This is because the recruitment rate of RVO is low, and it is extremely difficult to recruit treatment naïve RVO patients for the study. Secondly, the study measured complement proteins but did not test complement activity in the blood and aqueous humour. Third, the study was conducted in a single centre and the results can only reflect the biological features of RVO in the local ethical population. Replication of the study findings with a larger sample size and in multiple ethnic groups is necessary to confirm our results. However, it should be noted that single-centre study reduces procedure-related variation and increases the reliability of the results in small sample size studies.

In conclusion, our study suggests that RVO patients had higher levels of complement activation in the blood and aqueous humour. Intraocular complement proteins may participate in retinal oedema and microvascular remodeling in RVO. FDA has approved several complement inhibitors (e.g., Pegcetabopla, Eculizumab, etc.) for the treatment of various angioedema and autoimmune diseases [30]. Further study on the role of the complement system in RVO will help to determine whether these complement inhibitors can be repurposed for RVO management.

## Data Availability

All data generated or analyzed during this study are included in this article. Further enquiries can be directed to the corresponding author.

## Abbreviations

RVO: Retinal vein occlusion
BRVO: Branch retinal vein occlusion
CRVO: Central retinal vein occlusion
VEGF: Vascular endothelial cell growth factor
CP: The classical pathway
AP: The alternative pathway
MBL: Mannose-binding lectin
CFB: Complement factor B
CFD: Complement factor D
CFI: Complement factor I
CFH: Complement factor H
VA: Visual acuity
FAZ: Foveal avascular zone
PERIM: Perimeter of foveal avascular zone
CRT: Central retinal thickness
ILM-IPL: Inner limiting membrane-inner plexiform layer
SVD: Superficial capillary plexus vessel density
DVD: Deep capillary plexus vessel density
